# 6*H*,12*H*-5,11-Ethano­dibenzo[*b*,*f*][1,5]diazo­cine

**DOI:** 10.1107/S1600536808000883

**Published:** 2008-01-16

**Authors:** Masoud Faroughi, Andrew C. Try, Peter Turner

**Affiliations:** a Department of Chemistry and Biomolecular Sciences, Building F7B, Macquarie University, NSW 2109, Australia; b Crystal Structure Analysis Facility, School of Chemistry, F11, University of Sydney, NSW 2006, Australia

## Abstract

In the mol­ecule of the title compound, C_16_H_16_N_2_, the ethano-strapped analogue of unsubstituted Tröger’s base, the dihedral angle between the two benzene rings is 75.85 (4)°, the smallest angle measured for an ethano-strapped analogue.

## Related literature

For related literature, see: Hamada & Mukai (1996 ▶); Ishida *et al.* (2005 ▶); Solano *et al.* (2005 ▶); Faroughi *et al.* (2006*a* ▶,*b* ▶); Faroughi, Try & Turner (2007 ▶); Faroughi, Jensen & Try (2007 ▶). For related structures, see: Faroughi, Try, Klepetko *et al.* (2007 ▶); Faroughi *et al.* (2008 ▶).
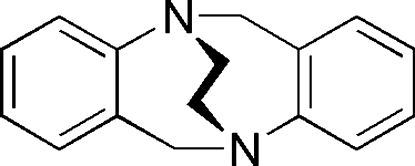


## Experimental

### 

#### Crystal data

C_16_H_16_N_2_
*M* *_r_* = 236.31
Orthorhombic, 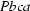

*a* = 11.717 (2) Å
*b* = 8.907 (2) Å
*c* = 22.829 (4) Å
*V* = 2382.5 (8) Å^3^
*Z* = 8
Mo *K*α radiation
μ = 0.08 mm^−1^
*T* = 150 (2) K
0.43 × 0.42 × 0.15 mm

#### Data collection

Bruker SMART 1000 CCD diffractometer
Absorption correction: Gaussian (Coppens *et al.*, 1965 ▶) and *XPREP* (Siemens, 1995 ▶) *T*_min_ = 0.968, *T*_max_ = 0.990
21723 measured reflections
2913 independent reflections
2398 reflections with *I* > 2σ(*I*)
*R*_int_ = 0.039

#### Refinement

*R*[*F* ^2^ > 2σ(*F* ^2^)] = 0.037
*wR*(*F* ^2^) = 0.099
*S* = 1.03
2913 reflections
163 parameters
H-atom parameters constrained
Δρ_max_ = 0.29 e Å^−3^
Δρ_min_ = −0.20 e Å^−3^

### 

Data collection: *SMART* (Siemens, 1995 ▶); cell refinement: *SAINT* (Siemens, 1995 ▶); data reduction: *SAINT* and *XPREP* (Siemens, 1995 ▶); program(s) used to solve structure: *SIR97* (Altomare *et al.*, 1999 ▶); program(s) used to refine structure: *SHELXL97* (Sheldrick, 2008 ▶); molecular graphics: *TEXSAN* (Molecular Structure Corporation, 1998 ▶), *Xtal3.6* (Hall *et al.*, 1999 ▶), *ORTEPII* (Johnson, 1976 ▶) and *WinGX* (Farrugia, 1999 ▶); software used to prepare material for publication: *WinGX*.

## Supplementary Material

Crystal structure: contains datablocks global, I. DOI: 10.1107/S1600536808000883/tk2243sup1.cif

Structure factors: contains datablocks I. DOI: 10.1107/S1600536808000883/tk2243Isup2.hkl

Additional supplementary materials:  crystallographic information; 3D view; checkCIF report

## Figures and Tables

**Fig. 1.**
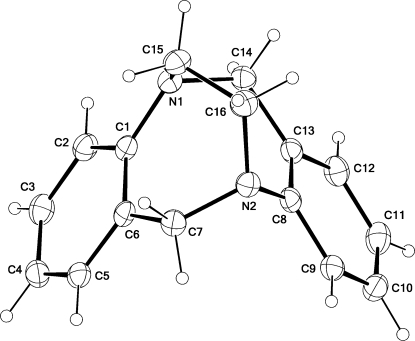
View of (I), showing the atomic numbering scheme. Displacement ellipsoids are shown at the 50% probability level.

**Fig. 2.**
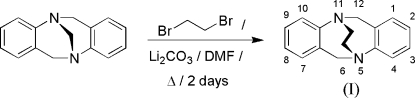
Synthetic scheme for the synthesis of (I) showing the numbering system used in naming the compound.

## supplementary crystallographic information

### Comment

Tröger's base compounds related to the title compound, (I), Fig. 1, are formed from an acid catalysed condensation of anilines, or a range of other amino aromatics, with either formaldehyde or formaldehyde equivalents. The compounds are characterized by the presence of a methano-strapped diazocine ring that is fused to two aromatic rings and this strapped ring system imparts a V-shaped structure on the compounds. The dihedral angle between the aromatic rings has been measured for over 20 simple dibenzo Tröger's base analogues and has been found to lie between 82° (Solano *et al.*, 2005) and 108° (Faroughi *et al.*, 2006*b*). It has been shown that reaction of 1,2-dibromoethane with several Tröger's base compounds affords ethano-straped analogues (Hamada & Mukai, 1996; Ishida *et al.*, 2005; Faroughi *et al.*, 2007*a*; Faroughi *et al.*, 2008), as outlined in Fig. 2. The structure of (I) is the third reported structure of an ethano-strapped analogue of Tröger's base. All three structures support the results of molecular modelling studies, which predict that the ethano-strapped analogues should have smaller dihedral angles in comparison with their methano-strapped precursors. The size of the angle for the methano-strapped structures (2,8-dibromo, 2,8-dichloro and unsubstituted, respectively) are as follows: 95° (Faroughi *et al.*, 2006*a*), 96° (Faroughi *et al.*, 2007*b*) and 95° (Faroughi, Jensen & Try, 2007), whilst the corresponding values for the ethano-strapped structures are 86° (Faroughi *et al.*, 2007*a*), 87° (Faroughi *et al.*, 2008) and, for the subject of this report, (I) 76°.

### Experimental

The title compound was prepared according to the literature procedure (Hamada & Mukai, 1996) in 37% yield. Single crystals were produced from slow evaporation of a dichloromethane solution of (I).

### Refinement

H atoms were positioned geometrically, with C—H = 0.95 and 0.99 Å for aromatic and methylene H atoms, respectively, and constrained to ride on their parent atoms, with *U*_iso_(H) = 1.2*U*_eq_(C).

### Crystal data

**Table:**

C_16_H_16_N_2_	*D*_x_ = 1.318 Mg m^−^^3^
*M**_r_* = 236.31	Melting point: 447 K
Orthorhombic, *P**b**c**a*	Mo *K*α radiation λ = 0.71073 Å
Hall symbol: -P 2ac 2ab	Cell parameters from 985 reflections
*a* = 11.717 (2) Å	θ = 2.5–27.9º
*b* = 8.907 (2) Å	µ = 0.08 mm^−^^1^
*c* = 22.829 (4) Å	*T* = 150 (2) K
*V* = 2382.5 (8) Å^3^	Plate, colourless
*Z* = 8	0.43 × 0.42 × 0.15 mm
*F*_000_ = 1008	

### Data collection

**Table:**

Bruker SMART 1000 CCD diffractometer	2913 independent reflections
Radiation source: fine-focus sealed tube	2398 reflections with *I* > 2σ(*I*)
Monochromator: graphite	*R*_int_ = 0.039
*T* = 150(2) K	θ_max_ = 28.3º
ω scans	θ_min_ = 1.8º
Absorption correction: Gaussian(Coppens *et al.*, 1965) and XPREP (Siemens, 1995)	*h* = −15→15
*T*_min_ = 0.968, *T*_max_ = 0.990	*k* = −11→11
21723 measured reflections	*l* = −29→28

### Refinement

**Table:**

Refinement on *F*^2^	Secondary atom site location: difference Fourier map
Least-squares matrix: full	Hydrogen site location: inferred from neighbouring sites
*R*[*F*^2^ > 2σ(*F*^2^)] = 0.037	H-atom parameters constrained
*wR*(*F*^2^) = 0.099	*w* = 1/[σ^2^(*F*_o_^2^) + (0.0474*P*)^2^ + 0.8505*P*] where *P* = (*F*_o_^2^ + 2*F*_c_^2^)/3
*S* = 1.03	(Δ/σ)_max_ < 0.001
2913 reflections	Δρ_max_ = 0.29 e Å^−^^3^
163 parameters	Δρ_min_ = −0.20 e Å^−^^3^
Primary atom site location: structure-invariant direct methods	Extinction correction: none

### Special details

**Table:**

Geometry. All e.s.d.'s (except the e.s.d. in the dihedral angle between two l.s. planes) are estimated using the full covariance matrix. The cell e.s.d.'s are taken into account individually in the estimation of e.s.d.'s in distances, angles and torsion angles; correlations between e.s.d.'s in cell parameters are only used when they are defined by crystal symmetry. An approximate (isotropic) treatment of cell e.s.d.'s is used for estimating e.s.d.'s involving l.s. planes.
Refinement. Refinement of *F*^2^ against ALL reflections. The weighted *R*-factor *wR* and goodness of fit *S* are based on *F*^2^, conventional *R*-factors *R* are based on *F*, with *F* set to zero for negative *F*^2^. The threshold expression of *F*^2^ > σ(*F*^2^) is used only for calculating *R*-factors(gt) *etc*. and is not relevant to the choice of reflections for refinement. *R*-factors based on *F*^2^ are statistically about twice as large as those based on *F*, and *R*- factors based on ALL data will be even larger.

### Fractional atomic coordinates and isotropic or equivalent isotropic displacement parameters (Å^2^)

**Table:**

	*x*	*y*	*z*	*U*_iso_*/*U*_eq_	
N1	0.12902 (7)	0.27164 (10)	0.17143 (4)	0.0194 (2)	
N2	0.04073 (7)	−0.01939 (10)	0.19142 (4)	0.0194 (2)	
C1	0.01598 (9)	0.29851 (11)	0.15021 (5)	0.0187 (2)	
C2	0.00104 (9)	0.42146 (12)	0.11285 (5)	0.0222 (2)	
H2	0.0648	0.4824	0.1031	0.027*	
C3	−0.10531 (10)	0.45585 (12)	0.08975 (5)	0.0244 (2)	
H3	−0.1138	0.5389	0.0640	0.029*	
C4	−0.19937 (9)	0.36838 (12)	0.10442 (5)	0.0229 (2)	
H4	−0.2729	0.3929	0.0897	0.027*	
C5	−0.18463 (9)	0.24492 (12)	0.14072 (5)	0.0202 (2)	
H5	−0.2489	0.1848	0.1503	0.024*	
C6	−0.07795 (9)	0.20660 (11)	0.16355 (4)	0.0184 (2)	
C7	−0.06746 (9)	0.06157 (12)	0.19875 (5)	0.0203 (2)	
H7A	−0.0772	0.0857	0.2408	0.024*	
H7B	−0.1307	−0.0061	0.1874	0.024*	
C8	0.07275 (9)	−0.04025 (11)	0.13126 (5)	0.0180 (2)	
C9	0.02315 (9)	−0.16067 (12)	0.10144 (5)	0.0218 (2)	
H9	−0.0295	−0.2238	0.1214	0.026*	
C10	0.04935 (10)	−0.18974 (13)	0.04328 (5)	0.0252 (2)	
H10	0.0139	−0.2711	0.0235	0.030*	
C11	0.12743 (10)	−0.09959 (13)	0.01412 (5)	0.0261 (3)	
H11	0.1471	−0.1200	−0.0255	0.031*	
C12	0.17656 (10)	0.02068 (13)	0.04333 (5)	0.0234 (2)	
H12	0.2298	0.0824	0.0232	0.028*	
C13	0.14986 (9)	0.05357 (11)	0.10154 (5)	0.0187 (2)	
C14	0.20158 (9)	0.19445 (12)	0.12853 (5)	0.0207 (2)	
H14A	0.2742	0.1667	0.1479	0.025*	
H14B	0.2200	0.2657	0.0966	0.025*	
C15	0.13809 (10)	0.20766 (12)	0.23032 (5)	0.0225 (2)	
H15A	0.0759	0.2483	0.2550	0.027*	
H15B	0.2116	0.2388	0.2479	0.027*	
C16	0.13116 (9)	0.03597 (12)	0.23015 (5)	0.0221 (2)	
H16A	0.2053	−0.0057	0.2172	0.027*	
H16B	0.1166	0.0001	0.2705	0.027*	

### Atomic displacement parameters (Å^2^)

**Table:**

	*U*^11^	*U*^22^	*U*^33^	*U*^12^	*U*^13^	*U*^23^
N1	0.0199 (4)	0.0179 (4)	0.0205 (4)	−0.0011 (3)	−0.0002 (3)	−0.0007 (3)
N2	0.0207 (4)	0.0183 (4)	0.0192 (4)	−0.0001 (3)	0.0008 (3)	0.0009 (3)
C1	0.0212 (5)	0.0159 (5)	0.0190 (5)	0.0006 (4)	0.0008 (4)	−0.0029 (4)
C2	0.0248 (5)	0.0167 (5)	0.0251 (5)	−0.0013 (4)	0.0030 (4)	0.0003 (4)
C3	0.0309 (6)	0.0186 (5)	0.0238 (5)	0.0033 (4)	0.0003 (4)	0.0022 (4)
C4	0.0224 (5)	0.0240 (5)	0.0222 (5)	0.0048 (4)	−0.0012 (4)	−0.0032 (4)
C5	0.0203 (5)	0.0207 (5)	0.0196 (5)	−0.0001 (4)	0.0030 (4)	−0.0041 (4)
C6	0.0216 (5)	0.0162 (5)	0.0175 (5)	0.0010 (4)	0.0030 (4)	−0.0032 (4)
C7	0.0208 (5)	0.0196 (5)	0.0207 (5)	−0.0008 (4)	0.0034 (4)	0.0014 (4)
C8	0.0176 (5)	0.0159 (5)	0.0206 (5)	0.0032 (4)	−0.0004 (4)	0.0016 (4)
C9	0.0198 (5)	0.0170 (5)	0.0284 (6)	0.0011 (4)	−0.0015 (4)	0.0005 (4)
C10	0.0282 (6)	0.0202 (5)	0.0270 (6)	0.0033 (4)	−0.0059 (4)	−0.0045 (4)
C11	0.0323 (6)	0.0260 (6)	0.0199 (5)	0.0074 (5)	0.0003 (4)	−0.0020 (4)
C12	0.0243 (5)	0.0227 (5)	0.0231 (5)	0.0034 (4)	0.0036 (4)	0.0029 (4)
C13	0.0173 (5)	0.0171 (5)	0.0216 (5)	0.0029 (4)	−0.0001 (4)	0.0013 (4)
C14	0.0180 (5)	0.0197 (5)	0.0244 (5)	−0.0014 (4)	0.0024 (4)	0.0006 (4)
C15	0.0253 (5)	0.0219 (5)	0.0204 (5)	−0.0018 (4)	−0.0028 (4)	−0.0012 (4)
C16	0.0245 (5)	0.0217 (5)	0.0202 (5)	−0.0002 (4)	−0.0026 (4)	0.0022 (4)

### Geometric parameters (Å, °)

**Table:**

N1—C1	1.4305 (14)	C8—C9	1.3970 (15)
N1—C15	1.4639 (14)	C8—C13	1.4053 (15)
N1—C14	1.4679 (13)	C9—C10	1.3873 (16)
N2—C8	1.4358 (14)	C9—H9	0.9500
N2—C16	1.4654 (14)	C10—C11	1.3874 (17)
N2—C7	1.4680 (14)	C10—H10	0.9500
C1—C2	1.3991 (15)	C11—C12	1.3870 (17)
C1—C6	1.4051 (15)	C11—H11	0.9500
C2—C3	1.3873 (16)	C12—C13	1.3964 (15)
C2—H2	0.9500	C12—H12	0.9500
C3—C4	1.3906 (16)	C13—C14	1.5236 (15)
C3—H3	0.9500	C14—H14A	0.9900
C4—C5	1.3877 (16)	C14—H14B	0.9900
C4—H4	0.9500	C15—C16	1.5315 (15)
C5—C6	1.3966 (15)	C15—H15A	0.9900
C5—H5	0.9500	C15—H15B	0.9900
C6—C7	1.5262 (15)	C16—H16A	0.9900
C7—H7A	0.9900	C16—H16B	0.9900
C7—H7B	0.9900		
			
C1—N1—C15	116.32 (9)	C10—C9—C8	121.17 (10)
C1—N1—C14	112.87 (8)	C10—C9—H9	119.4
C15—N1—C14	112.85 (9)	C8—C9—H9	119.4
C8—N2—C16	115.58 (8)	C9—C10—C11	119.81 (10)
C8—N2—C7	113.47 (8)	C9—C10—H10	120.1
C16—N2—C7	112.97 (8)	C11—C10—H10	120.1
C2—C1—C6	119.35 (10)	C12—C11—C10	119.34 (11)
C2—C1—N1	116.96 (9)	C12—C11—H11	120.3
C6—C1—N1	123.66 (9)	C10—C11—H11	120.3
C3—C2—C1	121.13 (10)	C11—C12—C13	121.78 (10)
C3—C2—H2	119.4	C11—C12—H12	119.1
C1—C2—H2	119.4	C13—C12—H12	119.1
C2—C3—C4	119.78 (10)	C12—C13—C8	118.60 (10)
C2—C3—H3	120.1	C12—C13—C14	117.94 (9)
C4—C3—H3	120.1	C8—C13—C14	123.39 (9)
C5—C4—C3	119.28 (10)	N1—C14—C13	115.16 (8)
C5—C4—H4	120.4	N1—C14—H14A	108.5
C3—C4—H4	120.4	C13—C14—H14A	108.5
C4—C5—C6	121.86 (10)	N1—C14—H14B	108.5
C4—C5—H5	119.1	C13—C14—H14B	108.5
C6—C5—H5	119.1	H14A—C14—H14B	107.5
C5—C6—C1	118.54 (10)	N1—C15—C16	112.49 (9)
C5—C6—C7	118.38 (9)	N1—C15—H15A	109.1
C1—C6—C7	122.97 (9)	C16—C15—H15A	109.1
N2—C7—C6	115.16 (8)	N1—C15—H15B	109.1
N2—C7—H7A	108.5	C16—C15—H15B	109.1
C6—C7—H7A	108.5	H15A—C15—H15B	107.8
N2—C7—H7B	108.5	N2—C16—C15	112.08 (9)
C6—C7—H7B	108.5	N2—C16—H16A	109.2
H7A—C7—H7B	107.5	C15—C16—H16A	109.2
C9—C8—C13	119.27 (10)	N2—C16—H16B	109.2
C9—C8—N2	117.18 (9)	C15—C16—H16B	109.2
C13—C8—N2	123.56 (9)	H16A—C16—H16B	107.9
			
C15—N1—C1—C2	147.34 (10)	C7—N2—C8—C13	97.36 (11)
C14—N1—C1—C2	−79.92 (11)	C13—C8—C9—C10	0.49 (16)
C15—N1—C1—C6	−34.51 (14)	N2—C8—C9—C10	−179.77 (10)
C14—N1—C1—C6	98.24 (12)	C8—C9—C10—C11	1.00 (16)
C6—C1—C2—C3	1.33 (16)	C9—C10—C11—C12	−1.34 (17)
N1—C1—C2—C3	179.57 (10)	C10—C11—C12—C13	0.20 (17)
C1—C2—C3—C4	0.89 (17)	C11—C12—C13—C8	1.28 (16)
C2—C3—C4—C5	−1.89 (16)	C11—C12—C13—C14	−175.68 (10)
C3—C4—C5—C6	0.67 (16)	C9—C8—C13—C12	−1.61 (15)
C4—C5—C6—C1	1.54 (15)	N2—C8—C13—C12	178.68 (9)
C4—C5—C6—C7	−174.80 (9)	C9—C8—C13—C14	175.18 (9)
C2—C1—C6—C5	−2.51 (15)	N2—C8—C13—C14	−4.53 (16)
N1—C1—C6—C5	179.38 (9)	C1—N1—C14—C13	−50.30 (12)
C2—C1—C6—C7	173.65 (10)	C15—N1—C14—C13	84.12 (11)
N1—C1—C6—C7	−4.46 (16)	C12—C13—C14—N1	146.11 (10)
C8—N2—C7—C6	−49.21 (12)	C8—C13—C14—N1	−30.70 (14)
C16—N2—C7—C6	84.85 (11)	C1—N1—C15—C16	86.36 (11)
C5—C6—C7—N2	144.50 (10)	C14—N1—C15—C16	−46.39 (12)
C1—C6—C7—N2	−31.66 (14)	C8—N2—C16—C15	86.95 (11)
C16—N2—C8—C9	144.83 (9)	C7—N2—C16—C15	−46.09 (12)
C7—N2—C8—C9	−82.36 (11)	N1—C15—C16—N2	−43.67 (13)
C16—N2—C8—C13	−35.45 (14)		
